# Diffusion kurtosis imaging as a biomarker of breast cancer

**DOI:** 10.1259/bjro.20220038

**Published:** 2023-01-14

**Authors:** Maya Honda, Denis Le Bihan, Masako Kataoka, Mami Iima

**Affiliations:** 1 Department of Diagnostic Imaging and Nuclear Medicine, Graduate School of Medicine, Kyoto University, Kyoto, Japan; 2 Department of Diagnostic Radiology, Kansai Electric Power Hospital, Osaka, Japan; 3 NeuroSpin, Joliot Institute, Paris-Saclay University, CEA-Saclay Center, Gif-sur-Yvette, France; 4 National Institute for Physiological Sciences (NIPS), Okazaki, Japan; 5 Institute for Advancement of Clinical and Translational Science, Kyoto University Hospital, Kyoto, Japan

## Abstract

Diffusion kurtosis imaging (DKI) is a diffusion-weighted imaging method that describes non-Gaussian signal behavior using a relatively simple mathematical model. A parameter, kurtosis K, describes the deviation of the diffusion signal decay from a Gaussian pattern. The deviation reflects the complexity of the tissue microstructure affecting water diffusion. Several studies have investigated the diagnostic performance of DKI in distinguishing malignant from benign breast lesions. DKI has been reported to correlate with subtypes and with several molecular and other factors related to the treatment and prognosis of breast cancer. Some technical considerations remain to be resolved for the clinical application of DKI in the breast.

**Advances in knowledge:**

DKI, which increases the sensitivity to complex tissue microstructure compared to standard DWI, has been applied in the breast, allowing to increase clinical performance in distinguishing malignant from benign lesions and in predicting prognosis or treatment response in breast cancer.

## Diffusion kurtosis imaging (DKI)

Diffusion-weighted imaging (DWI) has become widely used as a powerful adjunct in the evaluation of breast lesions.^1^ DWI can be assessed qualitatively and quantitatively, based on the standard apparent diffusion coefficient (ADC) introduced by Le Bihan et al.^2^ The ADC simplifies the processing of diffusion MRI data using a straightforward gaussian (monoexponential) approximation. Although water diffusion in tissues is mostly non-Gaussian, ADC values are typically lower in breast cancer than in benign breast lesions or normal breast tissues and provide the ability to detect breast lesions, distinguishing between malignant and benign, when used alone or combined with dynamic contrast-enhanced MRI.

**Figure 1.**
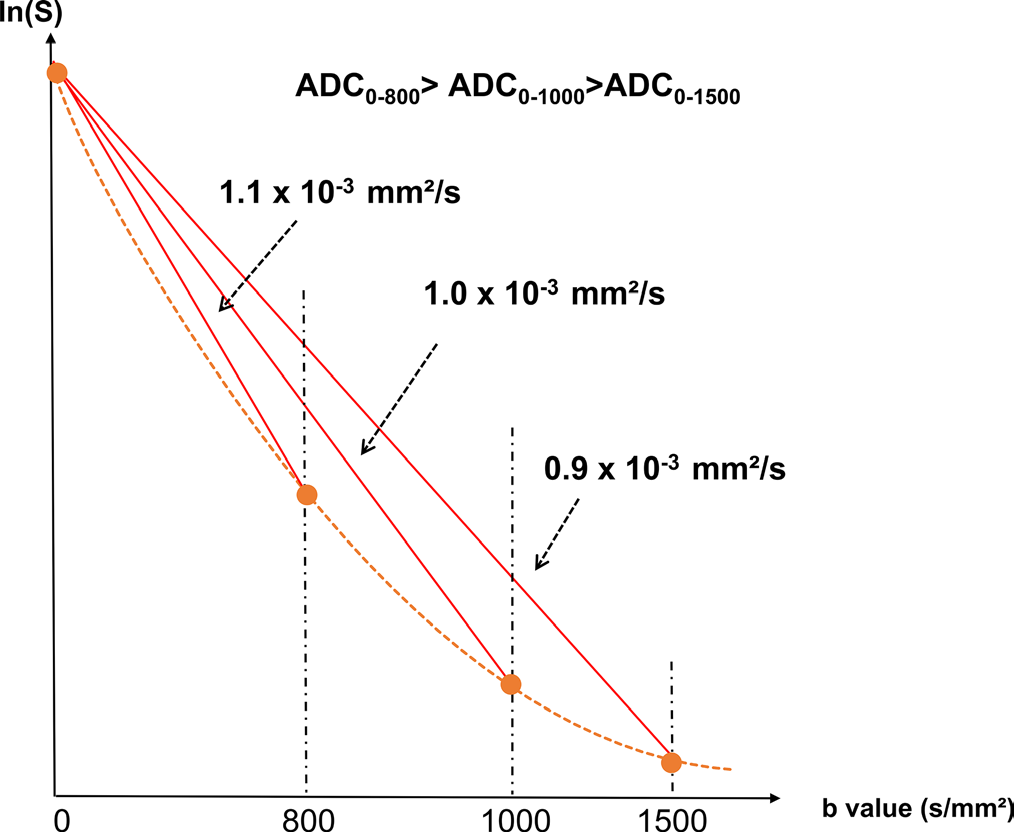
Example of signal decay and ADC values in typical breast cancer plotted against b values.

**Figure 2.**
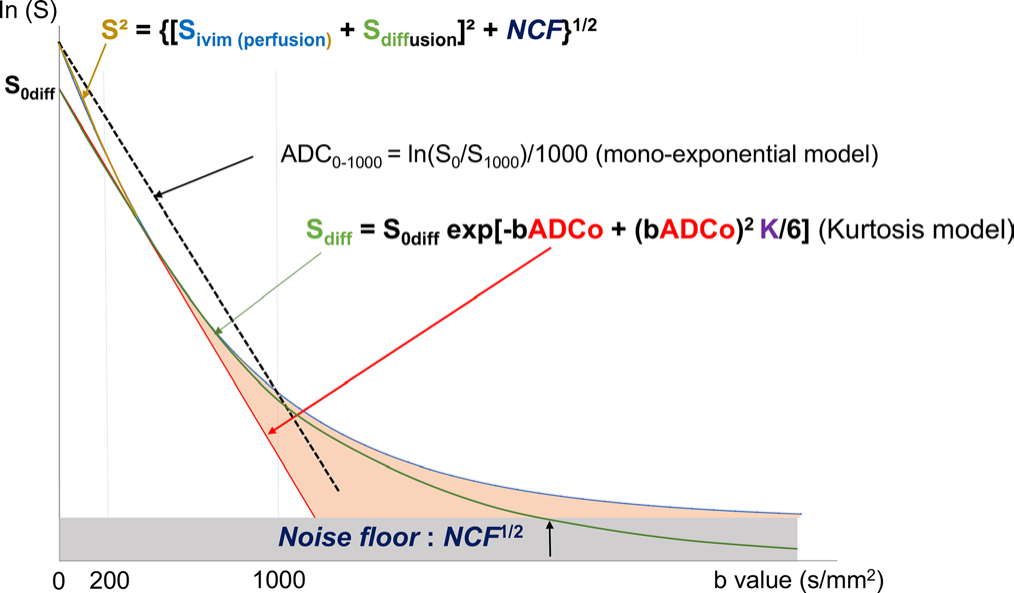
The raw signal can be fitted with a model, which includes perfusion-driven IVIM effects (very low b values), a tissue diffusion component, and noise floor (high b values). The kurtosis diffusion model has two parameters, ADC_0_ (theoretical ADC when b reaches 0, representing the Gaussian diffusion component [straight line] of the signal) and K. The ADC is obtained from the signals acquired at two b values (here, 0 and 1000 s/mm²) using a monoexponential (Gaussian) model. However, one can see that the ADC intrinsically includes IVIM, Gaussian, and non-Gaussian diffusion effects.

A drawback of DWI is that when using a high degree of sensitization to diffusion (so-called b values > 1000 s/mm²), the diffusion MRI signal deviates from assumed Gaussian behavior (Figure 1). This tendency can be observed as the ADC decreasing with the increased b value (Figure 2). While several models have been proposed to model this non-Gaussian signal behavior, the most popular approach to quantify this deviation is to rely on the ”excess kurtosis” or just “kurtosis”, which reflects the complexity of the tissue microstructure.^3,4^ The kurtosis can be estimated by developing the diffusion MRI signal decay with the b values as a polynomial to the second order (instead of one order for the ADC) (Figure 1).

DKI can be considered a purely mathematical model to describe the deviation of diffusion MRI signal decay without any particular biophysical assumptions about the underlying tissue structure. While DKI is relatively simple to implement compared to other techniques, such as the stretched exponential model and the biexponential model,^1^ it has intrinsic limitations. For instance, it does not fit the signal well when b values are >3000 s/mm². Also, it does not provide any specific information on the tissue microstructure (*e.g.,* cell density or size), which can be obtained using other specific models (such as neurite orientation dispersion and density imaging, NODDI, in the brain).^5^

**Figure 3.**
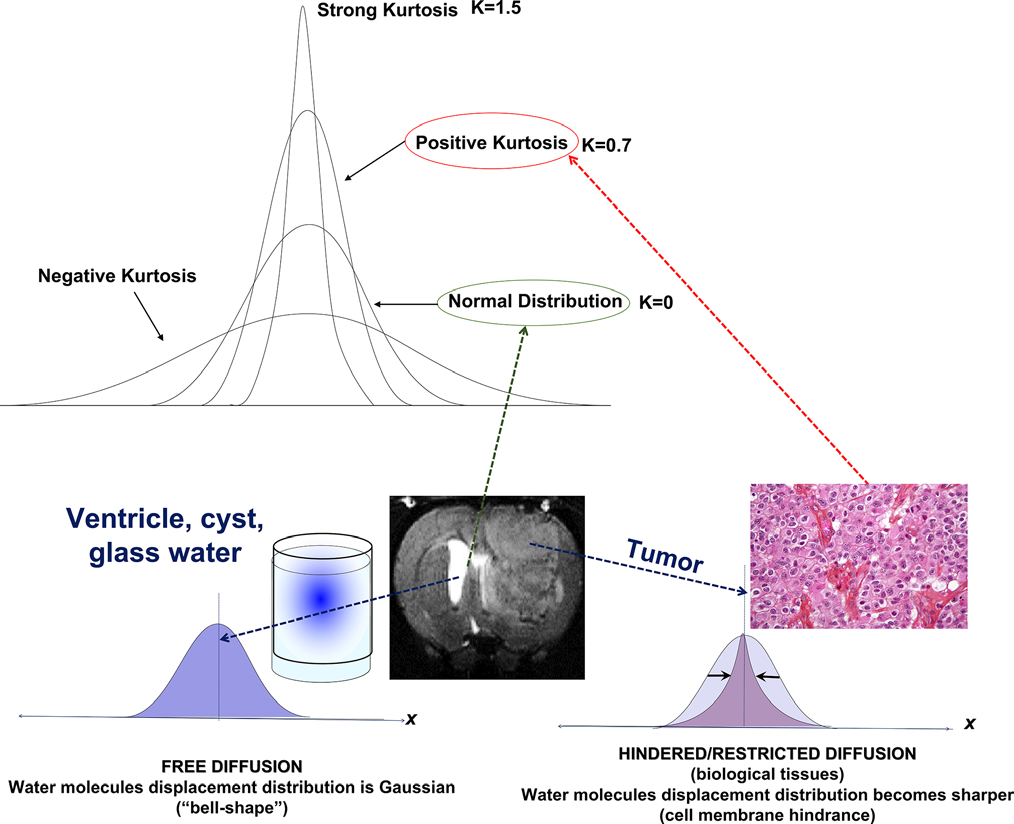
Kurtosis (**K**) describes the deviation of the diffusion signal decay from a Gaussian pattern. (**A**) When water diffuses freely, as in a glass or cyst, the random displacement of water molecules follows a Gaussian (normal) distribution (*K* = 0), the half-width of which is the diffusion coefficient. (**B**) In tumors with high cell density, water diffusion is hindered by molecular and cellular obstacles. As a result, the displacement distribution becomes sharper, and the diffusion coefficient decreases compared to free diffusion (*K* > 0). (Modified from Ref.6).

DKI provides a parameter, kurtosis, sometimes called K or mean kurtosis, MK, to describe the deviation of the diffusion signal decay from a Gaussian pattern. When we plot the probability, P(r,t), that a water molecule diffuses freely over a distance r in a time t, the resulting Gaussian distribution corresponds to *K* = 0. However, in the presence of non-Gaussian diffusion, the distribution becomes sharper, because the diffusion distance decreases, which also causes the signal to decay more slowly. This sharpness is quantified by a higher K value (Figure 3).^6^ The diffusion-weighted signal for a given b value, b, is then given by:

(1) $$S_{b}=S_{0}\cdot exp\left(-b\cdot {ADC}_{0}+\frac{1}{6}b^{2}\cdot {ADC}_{0}^{2}\cdot K\right)$$

where ADC_0_ is the ADC value extrapolated when b is close to 0 (pure Gaussian diffusion); in other words, the ADC with correction of the non-Gaussian bias. When *K* = 0, ADC_0_ becomes equal to ADC.

The application of DKI began with animal models, and then extended to clinical oncology (brain, prostate, squamous cell carcinoma, etc.) with studies on the breast appearing in 2014.^7^

## Clinical applications

### Distinction between malignant and benign breast lesions

**Figure 4.**
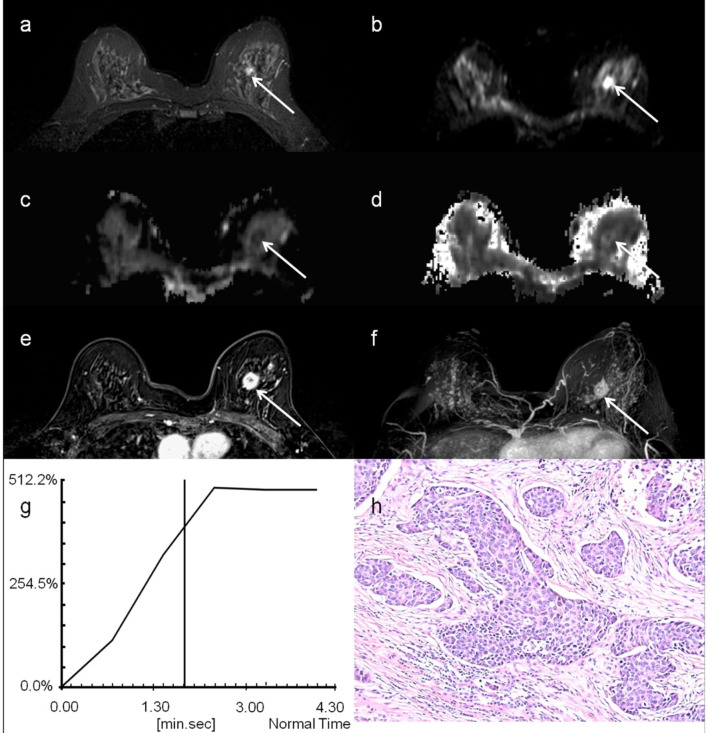
A 51-year-old female with infiltrating ductal carcinoma, indicated by the white arrow: (a) *T*_2_-weighted TSE image; (**b**) DW image at *b* = 0; (**c**) MD map; (**d**) MK map; (**e**) contrast enhancement map; (**f**) MIP of enhance map; (**g**) contrast enhancement curves and h) histological specimen. (Reproduced from Ref. 7).

Several studies so far have investigated the diagnostic performance of DKI in distinguishing malignant from benign breast lesions. Wu et al compared DKI parameters of 82 malignant and 42 benign breast lesions and found that K was significantly higher and ADC_0_ was significantly lower in malignant lesions than in benign lesions (Figure 4).^7^ Although other studies have also reported higher K and lower ADC_0_ values in breast cancer compared to benign breast lesions,^8,9^ this trend was not observed in another recent study.^10^ To resolve the inconsistency partly caused by a small sample size, meta-analyses have been published,^10,11^ Li and colleagues analyzed 13 studies including 867 malignant and 460 benign breast lesions. They found that breast cancer showed a higher K (standardized mean differences [SMD] = 1.23, *p* < 0.001) and a lower ADC_0_ (SMD = −1.29, *p* < 0.001) than benign lesions. The pooled sensitivity, specificity, and diagnostic odds ratio of K and ADC_0_ in distinguishing malignant from benign breast lesions (90%, 88% and 66 for K and 86%, 88% and 46 for ADC_0_) were higher than those of conventional ADC (85%, 83% and 29). In terms of AUC, however, K and ADC_0_ showed comparable diagnostic performance to that of ADC (0.90, 0.93 and 0.89, respectively).^11^

Among the 13 studies in the meta-analysis,^11^ three studies further investigated the use of K to distinguish invasive ductal carcinoma from ductal carcinoma *in situ* (DCIS). Lower K and higher ADC_0_ and ADC values were observed in DCIS than those in invasive carcinoma, and only K showed statistical significance in the pooled analysis (SMD = −1.36, *p* = 0.006).

The differences in K between breast cancer and benign lesions or DCIS may reflect the higher microstructural complexity in breast cancer. It should be noted that some point out that these kurtosis parameters were not overwhelmingly superior to conventional ADC in distinguishing between malignant and benign breast lesions.^12,13^ A recent study showed that the combination of K and ADC_0_ yielded higher discriminating power than K alone, but that the diagnostic performance was limited in non-mass breast lesions.^14^ Another study compared histogram parameters from DKI with those from DWI and found that the highest K value, Kmax, had the highest AUC, specificity, and accuracy, and that the AUC of the mean K value, Kmean, had significantly higher AUC than the mean ADC_0_ value and the mean ADC value, suggesting the possibility of better diagnostic performance to distinguish malignant from benign breast tumors than the conventional DWI model.^15^

**Figure 5.**
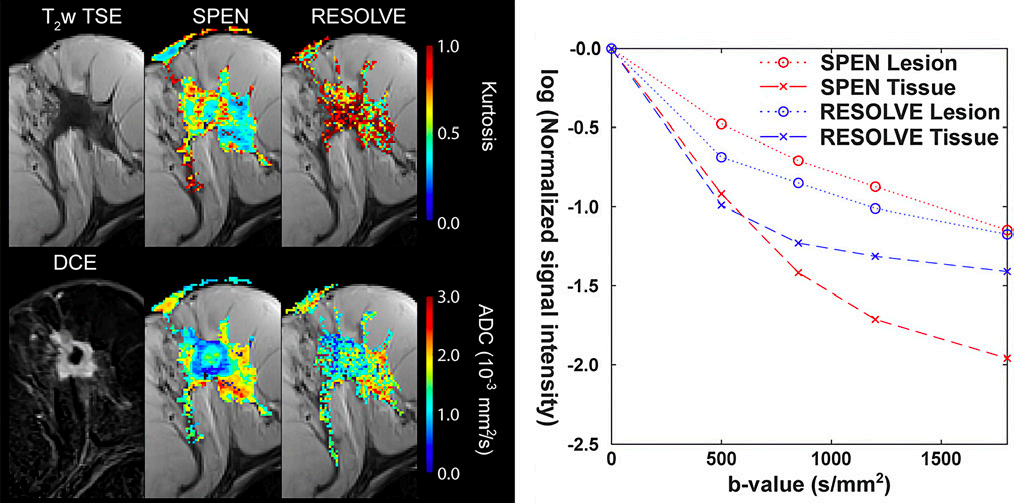
Summarizing SPEN’s and RESOLVE’s diffusion weighted results obtained for invasive ductal carcinoma. Shown are single-breast anatomical T2w (TSE) and T1w DCE subtraction images, with the latter highlighting as bright regions the cancerous masses. Shown as well are the ADC and kurtosis maps derived from SPEN and RESOLVE examinations (ADC maps were calculated solely based on images acquired with 0 and 850 s/mm^2^ nominal b values). The righthand panel summarizes the signal intensities segmented from the b-weighted SPEN and RESOLVE images, for regions identified as either healthy or cancerous tissue, based on the T2w and DCE images, respectively. Signal intensities integrated over these regions were then fitted; lines between points are shown as visual aids. Notice that since all areas were given an initial intensity of one, these points do not represent the images’ SNR. (Modified from Ref.17).

Because DKI is usually performed using echo-planar imaging (EPI) the voxel size is often large to maintain a good SNR, preventing the evaluation of small lesions.^16^ Some studies have limited the analysis to lesions larger than 5 to 10 mm. The use of readout-segmented EPI (RESOLVE) and spatiotemporal encoding (SPEN) may improve the image spatial resolution of DWI at high b values, which would be an advantage for the use of DKI for invasive breast cancer (Figure 5).^17^

### Association with subtypes and prognostic factors

Some studies have examined the value of DKI parameters for the detailed classification of breast cancer (Table 1). In terms of correlation with immunohistochemical findings, higher K in estrogen receptor (ER)-positive or progesterone receptor (PR)-positive cancers and lower K and lower ADC_0_ in human epithelial growth factor (HER) 2-positive cancer have been reported, although some reported inconsistent results.^8,14,18,19^

**Table 1.** Summary of K and ADC_0_ values in breast cancer by immunohistochemical status and proliferation rate

Indicators	Author	Year	Number of lesions analyzed	b-values (sec/mm^2^)	ER		PR		HER2		Ki-67	
					Positive	Negative	Positive	Negative	Positive	Negative	High (≥14%)	Low (<14%)
**K**	Sun et al. (8)	2015	52	0, 700, 1400, 2100, 2800	1.04 ± 0.17	1.14 ± 0.28	1.01 ± 0.14	1.11 ± 0.24	1.14 ± 0.30	1.03 ± 0.14	1.14 ± 0.23	0.96 ± 0.14
					*p* = 0.477		*p* = 0.190		*p* = 0.908		*p* = 0.001^*a*^	
	Iima et al. (29)	2018	140	5, 10, 20, 30, 50, 70, 100, 200, 400, 600, 800, 1000, 1500, 2000, 2500	0.86 (0.27)	0.84 (0.16)	0.87 (0.24)	0.84 (0.16)	0.84 (0.20)	0.86 (0.26)	0.85 (0.19)	0.86 (0.34)
					*p* = 0.11		*p* = 0.03*		*p* = 0.08		*p* = 0.75	
	Lu Yang et al. (14)	2021	212	0, 50, 1000, 2000	0.93 ± 0.20	0.81 ± 0.15	0.92 ± 0.20	0.83 ± 0.17	0.85 ± 0.16	0.92 ± 0.21	0.89 ± 0.18	0.90 ± 0.24
					*p* < 0.001^*a*^		*p* < 0.001^*a*^		*p* = 0.009^*a*^		*p* = 0.794	
	Meng et al. (18)	2021	76	0, 500,1000,1500, 2000	0.83 ± 0.09	0.87 ± 0.17	0.82 ± 0.09	0.87 ± 0.15	0.79 ± 0.08	0.89 ± 0.16	0.88 ± 0.13	0.88 ± 0.13
					*p* = 0.104		*p* = 0.152		*p* < 0.001^*a*^		*p* = 0.001^*a*^	
	Kang et al. (19)	2021	383	200, 500, 1000, 1500, 2000	1.163 (1.037, 1.268)	1.036 (0.940, 1.148)	1.160 (1.037, 1.268)	1.073 (0.949, 1.201)	1.120 (0.962, 1.212)	1.124 (0.997, 1.268)	1.134 (0.998, 1.249)	1.124 (0.997, 1.268)
					*p* < 0.001^*a*^		*p* < 0.001^*a*^		*p* = 0.077		*p* = 0.938	
	Wang et al. (20)	2022	130	0, 1000, 2000							0.76 ± 0.22	
											＜0.001^*a*^	
	Zhang et al. (25)	2022	81	0, 10, 30, 50, 100,150, 200, 500, 800, 1000, 1500, 2000, 2500	1.07 ± 0.18	0.95 ± 0.19	1.05 ± 0.18	0.95 ± 0.22	1.00 ± 0.22	1.03 ± 0.19	0.99 ± 0.18	1.087 ± 0.19
					*p* = 0.006^*a*^		*p* = .027^*a*^			*p* = 0.590	*p* = 0.024^*a*^ ^*b*^	
**ADC_0_**	Sun et al. (8)	2015	52	0, 700, 1400, 2100, 2800	1.09 ± 0.22	1.06 ± 0.22	1.11 ± 0.23	1.06 ± 0.22	1.05 ± 0.24	1.09 ± 0.21	1.02 ± 0.20	1.18 ± 0.22
					*p* = 0.774		*p* = 0.461		*p* = 0.667		*p* = 0.008^*a*^	
	Lu Yang et al. (14)	2021	212	0, 50, 1000, 2000	1.11 ± 0.25	1.20 ± 0.31	1.12 ± 0.27	1.17 ± 0.28	1.19 ± 0.31	1.10 ± 0.25	1.12 ± 0.27	1.20 ± 0.28
					*p* = 0.058		*p* = 0.154		*p* = 0.022^*a*^		*p* = 0.027^*a*^	
	Meng et al. (18)	2021	76	0, 500,1000,1500, 2000	1.16 ± 0.32	1.09 ± 0.41	1.15 ± 0.35	1.13 ± 0.37	0.79 ± 0.08	0.89 ± 0.16	1.04 ± 0.29	1.37 ± 0.41
					*p* = 0.427		*p* = 0.821		*p* < 0.001^*a*^		*p* = 0.002^*a*^	
	Kang et al. (19)	2021	383	200, 500, 1000, 1500, 2000	0.999 (0.882, 1.147)	0.992 (0.886, 1.128)	0.990 (0.879, 1.147)	0.999 (0.889, 1.143)	1.029 (0.911, 1.108)	0.988 (0.878, 1.176)	0.971 (0.875, 1.098)	1.033 (0.898, 1.199)
					*p* = 0.925		*p* = 0.573		*p* = 0.624		*p* = 0.005^*a*^	
	Wang et al. (20)	2022	130	0, 1000, 2000							2.41 ± 0.72	2.26 (1.91, 2.99)
											*p* = 0.984	
	Zhang et al. (25)	2022	81	0, 10, 30, 50, 100,150, 200, 500, 800, 1000, 1500, 2000, 2500	1.11 ± 0.27	1.22 ± 0.25	1.13 ± 0.27	1.22 ± 0.26	1.18 ± 0.26	1.16 ± 0.27	1.23 ± 0.24	1.03 ± 0.28
					*p* = 0.104		*p* = 0.205		*p* = 0.733		*p* = 0.002^*a*^ ^*b*^	

Data are mean values ± standard deviation or median values with interquartile range in parenthesis.

a statistically significant.

b A cut-off value is set on 20%.

In breast cancer subtypes, some studies have shown that the luminal subtype displays a higher K compared to non-luminal subtypes.^14,19^ However, higher ADC_0_ and lower K in the luminal subtype was reported in another recent study.^20^ This study also suggested improved diagnostic performance by combining DKI parameters with parameters obtained from dynamic contrast-enhanced MRI.

Histological grade and ki-67 labeling index are well-known prognostic factors in breast cancer. Some studies have reported that K correlates positively and ADC_0_ negatively with high histological grade and high ki-67 expression in invasive breast cancer.^8,14,18,19^

In a recent study using a virtual pathology technology to quantify the “area ratio” of the histological component to the entire tumor on pathology, K correlated with the “area ratio” of “cancer cell nuclei (*r* = 0.53, *p* = 0.00079)” among invasive ductal carcinoma masses, suggesting the utility of K in classification and grading breast tumors.^21^

In lymph node status, higher K and lower ADC_0_ values in positive lymph nodes were reported.^14,19,22^ Zhou et al reported borderline higher diagnostic efficiency of DKI compared with conventional MRI (*p* = 0.048).^22^

The 21-gene recurrence score (RS) has been routinely used to inform recommendations for chemotherapy among females with early-stage ER-positive, HER2-negative breast cancer. K, ADC_0_ and several histogram DKI parameters have been reported to show significant differences among low-, intermediate- and high-RS.^23,24^

## Treatment evaluation

Zhang et al evaluated the application of DKI in the evaluation of neoadjuvant chemotherapy among 81 breast cancer patients.^25^ In this study, K and ADC_0_ showed significant differences between pathological complete response (pCR) and non-pCR cases. After dividing the cohort into subgroups according to tumor phenotype, the same trend was observed only in the hormone receptor-positive and HER2-positive cancers.

A recent animal study showed interesting results from the use of kurtosis parameters for monitoring immunotherapy. Tang and colleagues compared an antiprogrammed death receptor-1(PD-1) therapy group with a control group in a triple negative breast cancer mouse model and found significant differences in K and ADC_0_ between the two groups at 5 and 10 days after treatment.^26^ Additionally, ADC_0_ was significantly different between the two groups at 15 days after treatment and correlated positively with histological CD45 levels, suggesting immune effects.^26^

### Association with prognosis

**Figure 6.**
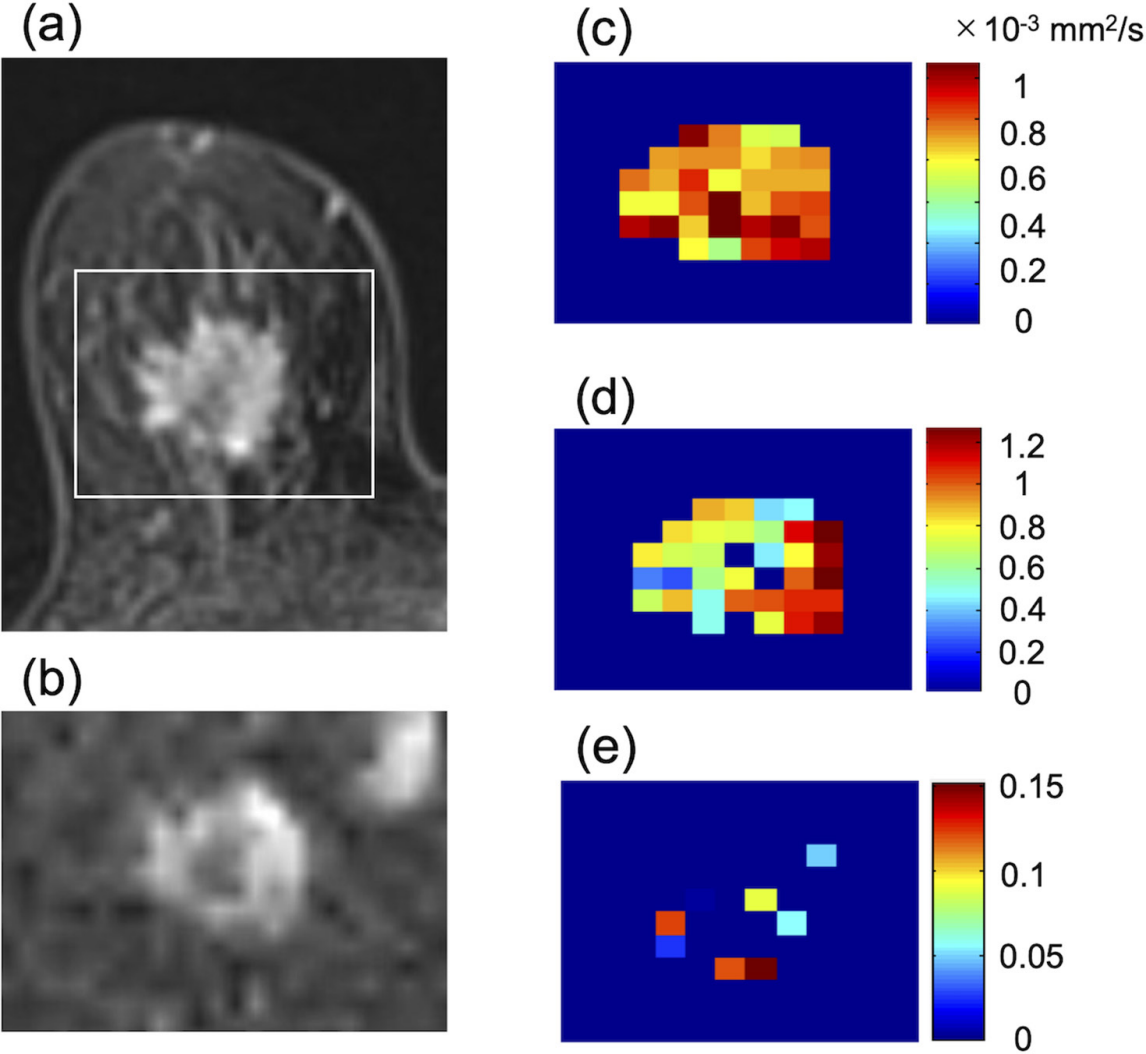
Images of a 77-year-old female with invasive ductal carcinoma who developed pleural metastases 5 years and 11 months after MR examination. (**a**) contrast-enhanced axial MR image, (**b**) DWI (*b* = 1500 s/mm^2^), (**c**) ADC_0_ map, (**d**) K map, (**e**) fIVIM map. The white rectangle on (**a**) shows the area covered by the parametric maps. (**a**) The mass lesion is heterogeneously enhancing. (**b**) The lesion shows heterogeneously high signal intensity on DWI. (**c**) ADC_0_ map shows low values throughout the lesion with a mean value of 0.9 × 10–3 mm^2^/s. (**d**) The mass shows relatively high mean K values (1.0) with heterogeneous distribution. (**e**) fIVIM map shows low values throughout the lesion with a mean value of<0.01. (Reprint from Ref. 27).

A recently published study analyzed whether DKI and intravoxel incoherent motion (IVIM) could predict the occurrence of distant metastases in 101 breast cancer patients. Significantly shorter distant disease-free survival was observed in group with higher K than in group with lower K (Figure 6). Other DKI- or IVM-derived parameters, pseudodiffusion (D^*^) and flowing blood volume fraction (fIVIM), did not affect outcome in their patient cohort.^27^

## Technical considerations

DKI requires the acquisition of DWI data with multiple b values, including relatively high values, and an algorithm to optimize the obtained signal to fit the formula (fitting). This imposes a tradeoff between the accurate estimation of kurtosis parameters and the total acquisition time when applying DKI to clinical practice. Indeed, while DKI has some clinical potential, as reviewed above, its poor robustness in acquisition remains a challenge, explaining perhaps the variability of the clinical outcomes. Several issues should be carefully considered in the acquisition and interpretation of DKI.

First, non-Gaussian processes can result from both the physical process itself, but also from intravoxel heterogeneity (non-mixing compartments) that cannot be distinguished from DKI which is a purely mathematical representation of the diffusion MRI signal. Imaging artifacts due to motion or geometric distortion, and thermal background noise (noise floor) often generate non-physiological kurtosis values during the fitting and lead to implausible (overestimated or, conversely, sometimes negative values). Correction of noise floor effect is necessary with low signal-to-noise ratio images (< 5), especially at high b values.^12^ In addition, in some circumstances, such as large K and high b values, the kurtosis model fails, as the theoretical DKI signal decay curve begins to increase with the b value, a physical impossibility, due to the limit of the polynomial decomposition of the signal decay to the second order (higher orders would then be necessary).

The least-squares method is the most common mathematical model fitting, which minimizes the residual values between the recorded data and the curve described by the fitted variables, although other approaches have been proposed. Fitting is sometimes performed as a two-step process when IVIM effects are present, first using the signal acquired with the highest b values to estimate ADC_0_ and K, then using those values with the complete signal sets to estimate the IVIM parameters. While voxel-by-voxel and volume-averaged signals can be used for fitting in the context of breast lesions in DKI,^28^ the volume- (or ROI-) averaged signals are expected to provide more accurate estimates than voxel-by-voxel signals.^29^

## Future expectations

The choice of b values (number and values, especially the highest values) varies considerably from study to study, which may have led to variations in the results. It is hoped that there will be some consensus on the protocols to use in the future. Meanwhile, one study compared the K values obtained from protocols with different b values (16 and 5), and from different numbers of excitations (NEX; 1 and 3) and found no significant differences. The results suggest that a limited MRI protocol using a few b values might be sufficient for the estimation of DKI parameters, facilitating its application in clinical settings in the breast.^30^

Regarding non-Gaussian diffusion, other approaches have been proposed without the need for modeling or fitting. The shifted ADC, sADC, is calculated from only two key b values, which intrinsically include non-Gaussian diffusion and IVIM effects.^31^ The S-index has been investigated as a model-free approach, which applies only two key b values to estimate both DKI and IVIM parameters utilizing a pre-built library of reference DWI signals typical of malignant and benign tissues.^32^ In this study, the diagnostic performance of the S-index combined with dynamic contrast-enhanced MRI using the Breast Imaging Reporting and Data System (BI-RADS) scoring were significantly higher than BI-RADS alone in terms of AUC, and combining the S-index and sADC yielded higher specificity than BI-RADS. High and significant correlations were identified with the S-index and prognostic factors, such as PR and HER2 status.^33^ These approaches may help balance the time and robustness of DKI.

## Conclusions

DKI has the potential to provide imaging parameters to reflect complexity in tissue microstructures using a relatively simple mathematical model. In the breast, several clinical applications have been explored, including distinguishing malignant from benign lesions and predicting prognosis or treatment response in breast cancer. Some technical considerations remain to be resolved for the clinical application of DKI in the breast.
